# Letter to editor about “Utilizing explainable machine learning for progression-free survival prediction in high-grade serous ovarian cancer: insights from a prospective cohort study”

**DOI:** 10.1097/JS9.0000000000003349

**Published:** 2025-09-08

**Authors:** Mengying Bai, Wenbo Wu, Yuehui Zheng

**Affiliations:** Shenzhen Traditional Chinese Medicine Hospital, The Fourth Clinical Medical College of Guangzhou University of Chinese Medicine, Shenzhen, People’s Republic of China

*Dear Editor,*

We read with great interest the recently published article entitled “Utilizing explainable machine learning for progression-free survival prediction in high-grade serous ovarian cancer: insights from a prospective cohort study*”*^[1]^. The authors should be commended for their efforts in developing an interpretable machine learning model to predict progression-free survival (PFS) in patients with high-grade serous ovarian cancer (HGSOC). While the study presents a commendable attempt at model construction, feature interpretation, and tool deployment, several methodological and conceptual issues warrant further discussion. This article was written following the TITAN guidelines^[2]^.

## Limited generalizability due to single-center study design

Although the study included 310 prospectively registered patients, all data were collected from a single tertiary hospital in China. The absence of external validation raises concerns about potential overfitting and restricts the model’s applicability in broader clinical settings. Given the heterogeneity of ovarian cancer across populations with different genetic backgrounds, healthcare systems, and treatment standards, validation using independent, multicenter cohorts is essential to assess the model’s robustness and transferability.

## Omission of molecular and imaging modalities limits biological and clinical insight

The study did not incorporate key molecular features known to influence HGSOC prognosis, such as BRCA1/2 mutation status, MSI, MMR deficiency, TP53 variant type, and tumor mutation burden (TMB)^[3]^. These biomarkers not only contribute to prognostic stratification but also guide treatment decisions – particularly regarding PARP inhibitor responsiveness. The lack of molecular subtyping limits the model’s biological interpretability and clinical relevance. Additionally, preoperative imaging data (e.g., CT, MRI) were not included. Given their importance in assessing tumor burden, lymph node involvement, and peritoneal spread, omitting imaging undermines the model’s ability to integrate visual disease indicators. With the advancement of multimodal AI, combining radiomics and clinical variables has been shown to enhance predictive performance^[4]^. Future models should consider integrating such data sources.

## Lack of comparison with established clinical scoring systems

Although the proposed model reportedly outperformed a traditional nomogram based on Cox regression (C-index ~ 0.65–0.71), it was not compared against commonly used clinical stratification tools such as FIGO staging or CA125 dynamics, nor against contemporary AI models^[5]^. Including these comparisons would help contextualize the model’s practical utility.

## Interpretability remains superficial without clinical depth

The study’s use of SHAP values to explore feature contributions is a welcome step toward interpretability. However, the clinical implications of these findings remain underdeveloped. For example, while HE4 was identified as a more influential variable than FIGO stage, the authors did not clarify whether this association is independent or consistent across early- and late-stage patients.

In summary, while the proposed framework represents a promising advancement in the prognostication of HGSOC, we encourage the authors to further validate their model using multicenter cohorts enriched with imaging and multi-omics data. Subgroup analysis, particularly in BRCA-mutated populations, may help improve model precision. Future work might also explore combined or dynamic endpoints (e.g., recurrence and overall survival) to better reflect real-world clinical needs. Making the model accessible through international platforms could also facilitate broader validation and iterative improvement.

## Data Availability

Not applicable.
